# Automatic Detection of Driver Fatigue Using Driving Operation Information for Transportation Safety

**DOI:** 10.3390/s17061212

**Published:** 2017-05-25

**Authors:** Zuojin Li, Liukui Chen, Jun Peng, Ying Wu

**Affiliations:** College of Electrical and Information Engineering, Chongqing University of Science and Technology, Chongqing 401331, China; cqustclk@126.com (L.C.); pengjun70@126.com (J.P.)

**Keywords:** steering wheel angles (SWA), yaw angles (YA), approximate entropy (ApEn), fatigue detection, back propagation (BP) neural network

## Abstract

Fatigued driving is a major cause of road accidents. For this reason, the method in this paper is based on the steering wheel angles (SWA) and yaw angles (YA) information under real driving conditions to detect drivers’ fatigue levels. It analyzes the operation features of SWA and YA under different fatigue statuses, then calculates the approximate entropy (ApEn) features of a short sliding window on time series. Using the nonlinear feature construction theory of dynamic time series, with the fatigue features as input, designs a “2-6-6-3” multi-level back propagation (BP) Neural Networks classifier to realize the fatigue detection. An approximately 15-h experiment is carried out on a real road, and the data retrieved are segmented and labeled with three fatigue levels after expert evaluation, namely “awake”, “drowsy” and “very drowsy”. The average accuracy of 88.02% in fatigue identification was achieved in the experiment, endorsing the value of the proposed method for engineering applications.

## 1. Introduction

As reported by the World Health Organization, car accidents kill more than 1.3 million people worldwide every year [1], of which fatigued driving is a major cause. According to other statistics, it causes thousands of automobile crashes [2] and about 35–45% of vehicle accidents [3,4]. Fatigued driving usually means the disorder of mental and physical functions after a long-lasting drive, subsequently leading to a weakening of the driver’s ability to control the vehicle. The technology in automatic detection of driver fatigue under real driving conditions is meaningful for reducing road accidents caused by fatigued driving.

The existing detection systems for driver fatigue, according to the source of the surveillance data, fall into two categories: intrusive and non-intrusive. Intrusive systems use physiological data of drivers and analyze their rules of change during the driving process, so as to monitor the drivers’ fatigue status. These data mainly include electroencephalogram (EEG) [5,6,7,8], electrooculogram (EOG) and electrocardiograph (ECG) [9,10,11,12]. When driving on a real road, data collecting sensors are mounted on the driver’s body, which may distract him or make him uncomfortable. This greatly restricts the application of these methods in engineering. Non-intrusive systems acquire information about the vehicle or the driver without contact with the driver, so the monitoring process will not interfere with the driver. The data retrieved can better reflect the real behaviors of the driver and the real-time status of the vehicle [13]. Presently, the latter has become a hot topic in driver fatigue detection research [14,15,16]. The system based on SWA and YA is an example of non-intrusive methods.

Literature shows, after long duration driving, due to drowsiness and flagging energy, the driver’s ability to operate and control the vehicle will decline substantially. This subsequently will decrease the accuracy and frequency of the turning of the steering wheel [17,18,19,20]. The driver fatigue status can be effectively detected by collecting and analyzing SWA data, and constructing an identification model.

Research on drivers’ operation features have obtained valuable results. Fukuda [21], after analyzing the statistical features of SWA time series, found that the cyclicity of time series is inherently related to fatigue status. He discovered the on-line detection of driver fatigue levels, with an accuracy of 76–88%. Andarian [22] analyzed the change rules of drivers’ steering data, established a model and method for monitoring driver fatigue status. His method demonstrated an accuracy of 85%. Additionally, Bo et al. [14] used statistical analysis of SWA data, extracted 11 feature indexes reflecting drivers’ fatigue level, and built an SVM based fatigue classification and identification model. The three-level fatigue detection system boasts an accuracy of 87.7%. Unfortunately, most research is conducted in a driving simulator, and as a result, the validity in real road conditions remains unproven.

Bittner [23] reported the validity of fatigue features under real road conditions. According to this study, the standard deviation of steering angles, which saw good performance in a simulation environment, does not behave well for fatigue detection on a real road. The reason for this is that the steering features during real road driving relates not only to the driver’s fatigue status, but also the speed, driving habits and ability, and the road conditions. Meanwhile, on the real road, the stochastic jiggling due to uneven pavement will mix noise with the driver operation data or vehicle status data, and greatly increase the probability of drifting. Therefore, the analysis, selection and performance optimization of steering or yaw angles under real driving conditions become more complex.

This paper presents a fatigue driving detection system using SWA and YA information. At first, it analyzes the operation features demonstrated with SWA and YA signals at different fatigue levels. Then, it uses the non-linear feature theory of dynamic time series to calculate the ApEn features of fixed window SWA and YA time series. At last, with the fatigue features as input, it designs a “2-6-6-3” multi-level BP Neural Networks classifier for the detection of three fatigue levels during the driving test.

The rest of this article is structured in the following manner. Section 2 analyzes drivers’ operation characteristics in steering wheel angles and yaw angles. Section 3 presents the designed fatigue level classification method with BP Neural Networks classifier based on the ApEn features for a varying nonlinear parameter to measure the irregularity of steering wheel angles and yaw angles. Section 4 demonstrates the experiment and results on SWA and YA datasets collected from subjects under real road driving conditions. Discussion and a summary are presented in Section 5 and Section 6, respectively.

## 2. Analysis of Drivers’ Operation Characteristics

The driving process is a typical non-linear dynamic system. Exploring the non-linear features of the time series of the operation parameters is helpful to analyze and identify drivers’ fatigue status. In real driving conditions, drivers, to ensure the safety, should judge the vehicle status constantly during the driving process and make modifications if deviation occurs. When drivers are tired, their ability to perceive the environment, in order to determine the situation and to keep the vehicle in control will be diminished. This will lead to a greater number of errors, and lower accuracy in control. The controlled and status variables of the vehicle will show different fluctuation ranges or frequency. Previous research [14,15,21,24] shows that tired drivers will show obvious abnormal features in vehicle operation. For instance, the fluctuation range, frequency and speed of SWA and YA all show deviation to some extent. It is safe to say that drivers’ operation features reflect their fatigue status, and the non-linear features and irregularities expressed by SWA and YA data vary widely according to drivers’ fatigue levels.

**Figure 1:**
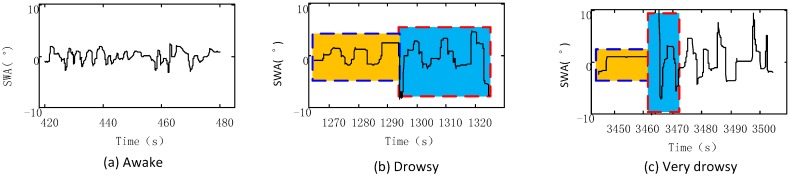
SWA waveforms under different fatigue statuses: (**a**) Awake; (**b**) drowsy; and (**c**) very drowsy. SWA: Steering wheel angles.

**Figure 2:**
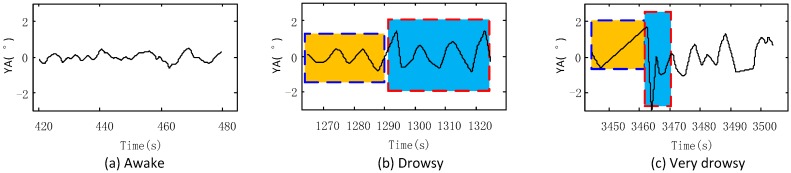
YA waveforms under different fatigue statuses: (**a**) Awake; (**b**) drowsy; and (**c**) very drowsy. YA: Yaw angles.

Waveforms in Figure 1 and Figure 2 are used to visually express how the driver’s fatigue levels affect his operation features. Figure 1 shows the SWA waveforms at different fatigue levels, from which we can see that when the driver is sober, as shown in Figure 1a, he will modify the steering wheel angles frequently in a small range. When he is tired, as shown in Figure 1b, the frequency of modification is low, as indicated by the waveforms in the yellow block, while the modification amplitude becomes larger and velocity higher, indicated in the blue block. If the driver is severely tired, as shown in Figure 1c, steering wheel angles remain unchanged for a period of time, as indicated by the yellow block, followed by quick fluctuations with big amplitude, as indicated by the blue block.

Figure 2 shows the typical YA waveforms under different fatigue levels. We can see that when the driver is sober, as shown in Figure 2a, the waveforms fluctuate frequently in a small range. When he is tired, as in Figure 2b, the fluctuations slow down as in the yellow block, and the amplitude and velocity increase as shown in the blue block. When the driver is extremely tired, as shown in Figure 2c, the waveforms become sharp when the SWA remains unchanged, as under the yellow block, and then quick and huge fluctuations occur as the SWA modification become quick and substantial, as under the blue block.

To summarize, the drivers’ operation features under fatigue mainly lie in the amplitude, velocity, and frequency of the changing SWA and YA parameters. As his fatigue grows, the driver’s steering modifications will decline in frequency, rise in amplitude, add in velocity, and even remain still for a period of time. The YA parameters also demonstrate features of slow frequency, large amplitude and high velocity.

## 3. Methodology

### 3.1. Fatigue Detection Scheme

**Figure 3:**
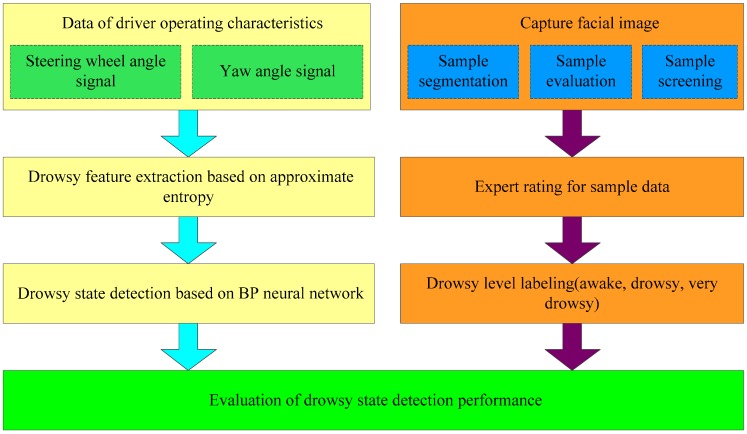
Framework of fatigue detection system.

Figure 3 is the framework of the driver fatigue detection algorithm. First of all, the fatigue driving experiment is conducted to acquire necessary datasets, which are processed with the Facial Video Expert Evaluation Method to produce graded fatigue data. Then, operation features of drivers at different fatigue grades are analyzed and the ApEns of SWA and YA time series are calculated to explore the non-linear features of these operation parameters. Finally, a three-level fatigue online detection algorithm is constructed based on the BP Neural Network to complete the driver fatigue detection.

### 3.2. Criteria of Fatigue Level Evaluation

In this paper, we define three fatigue levels: awake, drowsy, and very drowsy. Before evaluating the validity of the fatigue detection method, we should design a set of criteria to determine the fatigue level of the data, which serves as the standard patterns for the identification of fatigue levels. The facial video expert evaluation is, up to now, the most applicable method for driver fatigue status identification. This method requires a group of well-trained experts to score the fatigue status of drivers according to their facial expressions, head positions, and other factors. Wierwill et al. [25] were the first to introduce this method in driver fatigue appreciation. To be specific, this method works as follows: after the drivers’ facial videos are segmented, experts score the video clips in random order, according the fatigue features like rubbing eyes, scratching faces, yawning, closing eyes, and adjusting body positions. The scores range from 0 to 100, and the average of the scores given by the experts for a certain clip is taken as its score. In application, however, the problems of the method used by Wierwill et al. lie in: (1) the criteria between different levels are not easily distinguishable, (2) reasons and methods are not given as to why and how the continuous scores are grouped into three discrete levels, and (3) they fail to compare the video evaluation method in time series with that in random series. Aiming at these problems, Bo et al. [26] revised the evaluation criteria for driver fatigue, as shown in Table 1. They also verified this method with the statistical consistency between the methods in time series and random series. This paper uses the facial video expert evaluation method with the criteria shown in Table 1 to mark drivers’ three-level fatigue.

### 3.3. Extraction of ApEn of Time Series of the Operation Parameters

Non-linear property estimation is a complex analysis especially on nonlinear distributions. Approximate Entropy measures irregularity of time series data, and it is a non-linear dynamic parameter. In order to present the complexity of a non-linear time series and reflect the occurrence probability of a new pattern or regulation, ApEn assigns an estimated value of a non-negative fixed quantity. In previous work on complexity, algorithms of ApEn have been proven as a powerful estimator, with properties of usability and robusticity for irregularity or predictability evaluation. Studies [27,28,29] show that the changes of physiological state of the human body can be characterized by ApEn. ApEn has been very popular and applied to many static methods to quantify complexity and regularity. It shows great potential applications to analyze a wide variety of physiological and unphysiological time series data [30,31,32]. The ApEn of SWA and YA time series can be used to explore the irregularity of the driving process. By comparing the change and distribution of ApEn under different fatigue statuses, we can identify the fatigue level of drivers.

We can use time series data to obtain a robust estimate of ApEn. Consider an inputted time series, {u(n)=u(1),u(2),u(3),⋯,u(N)}, ApEn is calculated in Equation (1):

(1) $$ApEn(m,r,N)=\frac{1}{N-m+1}\sum_{i=1}^{N-m+1}\log(C_{i}^{m}(r))-\frac{1}{N-m}\sum_{i=1}^{N-m}\log(C_{i}^{m+1}(r))$$

where, $C_{i}^{m}(r)$ can be obtained through Equation (2).

(2) $$C_{i}^{m}(r)=\frac{B_{i}}{N-m+1}$$

Here, $B_{i}$ represent the number of j for example {d|X(i),X(j)|≤r}, and both X(i) and X(j) are used to represent inputted time series u(n)’s m-dimensional vectors reconstruction with Equation (3).

(3) $$X(i)=\left[u(i),u(i+1),\cdots \;,u(i+m-1)\right],\;X(i)\in R^{m}\;X(j)=\left[u(j),u(j+1),\cdots \;,u(j+m-1)\right],\;X(j)\in R^{m}$$

where d|X(i),X(j)| measures the distance between X(i) and X(j), i,j=1~N−m+1. To define the maximum difference between corresponding elements, variable m is set as an embedding dimension, r is the tolerance parameter as a scale, and N is the amount of time points in phase space. To calculate ApEn of SWA and YA data, with the increase of the value of m dimensions of embedded vectors and engineering computing workload, the change of wheel angles will be weakened at the same time, which will also weaken drowsiness features in SWA and YA data. On the other hand, with reference to the parameter value in ApEn applications suggested by Yentes [33], this paper chose m=2, and, as suggested by Pincus [34], the parameters r are typically chosen as r=0.2×SD, (*SD* represents standard deviation of the original windowing time series).

As shown in the above equation, in the calculation of the ApEn of time series, vectors should be circularly constructed to acquire the distances between them, which consume tremendous computing resources. Usually, construction of a distance matrix is used in engineering applications to reduce the consumption of resources. Equation (4) expresses a N×N distance matrix.

(4) $$d=\left|\left[u(n)\;u(n)\;\cdots u(n)\right]-\left[\begin{array}{c} u(n{)}^{\prime }\\ \vdots \\ u(n{)}^{\prime } \end{array}\right]\right|$$

### 3.4. Detection of Driver Fatigue Based on BP Neural Network

Established on the basis of modern biological research, an Artificial Neural Network (ANN) is a computation structure imitating the biological process to reflect some properties of human brains, and has been widely applied in intelligent information processing [35,36,37]. ANN classification is a typical statistical machine learning method, which requires no statistical properties or priori knowledge in the sample correlation domain. ANN is characteristic of high precision and efficiency in identification, which justifies its application in driver fatigue identification in this paper.

ANN, as a mathematical theory model imitating human brains or behaviors, is a non-algorithmic, non-linear and self-adaptive system, which is composed of a huge amount of computation processing units properly connected. Neuron is one unit can be expressed as:

(5) A=f(WP+B)

where W is the weight vector of ANN, B, threshold value vector of the network, P, input vector, and f transmission function.

Usually, neural networks need training rules, which involves constant adjustment of their link weight to minimize the error between the ideal and real outputs. Training is used to establish non-linear mapping between performance parameters and corresponding operation condition values. Neural network training needs to adjust weight W and threshold value B through sample learning, which is done by a specific training algorithm. The development of training algorithms is based on learning rules, which simulate biological learning mechanisms. Up to now, several types of neural networks with different structures have been reported. This paper uses the Multi-Layer Perception Neural Network (MLPNN) to realize the classification and identification of driver fatigue. 

**Figure 4:**
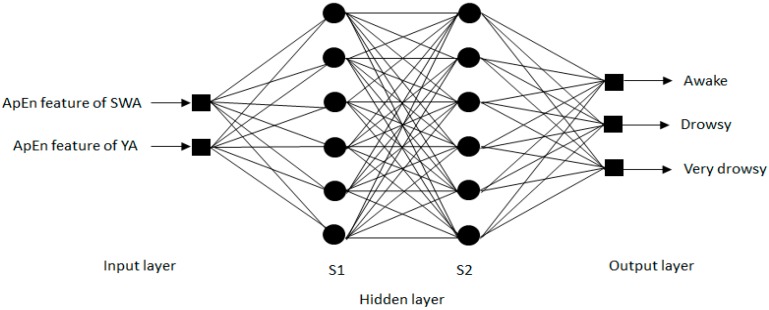
BP Network architecture.

MLPNN consists of one input layer, one or more hidden layers, and one output layer. This paper adopts a four-layer network to identify drivers’ fatigue status; namely, one input layer, two hidden layers and one output layer. The ApEn features of SWA and YA during the driving process are the input of the neural network, while the drivers’ fatigue levels, namely “awake”, “drowsy” and “very drowsy”, are the output. Different from input and output layers, there is no scientific and widespread method to determine the quantity of nodes in the hidden layers. If the node quantity is too low, the network cannot show the difference between different models, and let the fitting results approach to a linear relation. If the node quantity is too large, overfitting may occur in the network and lower the generalizability of untrained data. Moreover, with the increase in node numbers, the time required in computing will surge, making the engineering application difficult. Usually, the most popular way to determine the number of nods is conducting an experiment. We determine, through experiments, that the node numbers in the hidden layers are S1=6 and S2=6. Figure 4 shows the designed ANN structure, and other parameters of the network are set as: 0.082 for adaptive learning coefficient, 0.95 for momentum coefficient, 0.0001 for MSE, and tangent sigmoid for the activation function.

## 4. Experiments and Results

### 4.1. Experiment Setup

**Figure 5:**
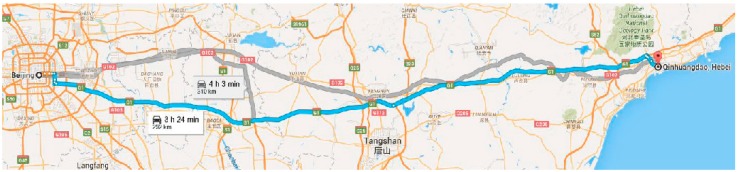
The driving route between Beijing and Qinhuangdao is shown on the blue line.

To obtain the driver fatigue level evaluation and experiment data, this paper selects the expressway from Beijing to Qinhuangdao as the driving route. The geographic environment is shown in Figure 5. The data collecting platforms in the experiment vehicles gather information for SWA, can_braking, can_thrott, brakeforce, leftsteer, rightsteer, yaw angle, X-accel, Y-accel, and synchronized drivers’ faces video. The participant drivers all possess a valid driver license and driving experience of more than one year. The experiment starts at noon when people are prone to drowsiness. A fifteen-minute trial experiment prepares the drivers for the operational environment, followed by the ninety minute formal experiment. The drivers’ task is to drive at approximately 100 km/h in the middle lane. Speed and position are kept according to the real driving conditions and their own driving habits. The length of the experiment is determined on the grounds that usually a driver becomes tired after one hour of driving in a monotonous driving environment, as revealed in previous experiments. Ninety minutes of driving can witness the whole process from “awake”, through “drowsy”, to “very drowsy”.

In a quiet driving environment, cameras mounted in the driving cabins record the drivers’ facial expressions, at a frequency of 15 Hz. The data collection systems in the vehicles record the status parameters mentioned above. However, as the SWA and YA can reflect drivers’ operation features more directly than other parameters, they contribute more to driver fatigue level identification. This paper chooses the two parameters from them as the objects in fatigue driving research, with the sampling frequency of 100 Hz. Ten drivers in total participated in this experiment, with an average age of 28 and 4.3 years of driving experience. The data acquired accumulated to 14.68 h.

### 4.2. Experiment Database

Whether to study the operation features of drivers in different fatigue statuses, or to design or build driver fatigue detection models, requires a set of sample data with known driver fatigue levels. For this reason, a reliably accurate measurement criterion is necessary to estimate the real fatigue levels of the drivers. After evaluation, the data samples with determined fatigue levels constitute the fatigue sample database.

In this paper, data collected in the experiment was processed with sample segmentation, evaluation, and screening to construct the driver fatigue sample database. In sample segmentation, the facial videos and operation features are segmented in synchronism. The former are segmented into one minute clips by software in chronological order, according to start time and end time. The operation feature data are also segmented. The two segments consist of one sample datum. The sample evaluation is conducted with the facial video expert evaluation method, with criteria shown in Table 1. Every facial video sample is scored in time series by three experts. The experts’ unanimous score is the determined fatigue level of a sample, and in case of disagreement between experts, three experts should negotiate. After negotiation, if agreement is reached, the result is taken as the fatigue level of the sample. If no agreement can be reached, this sample should be discarded. Additionally, samples involving a curved road or lane shift should also be discarded. Finally, the sample datasets labeled with fatigue levels consist of the three-level driver fatigue sample database, the composition of which in Table 2 is shown below:

### 4.3. Experiment Results

**Figure 6:**
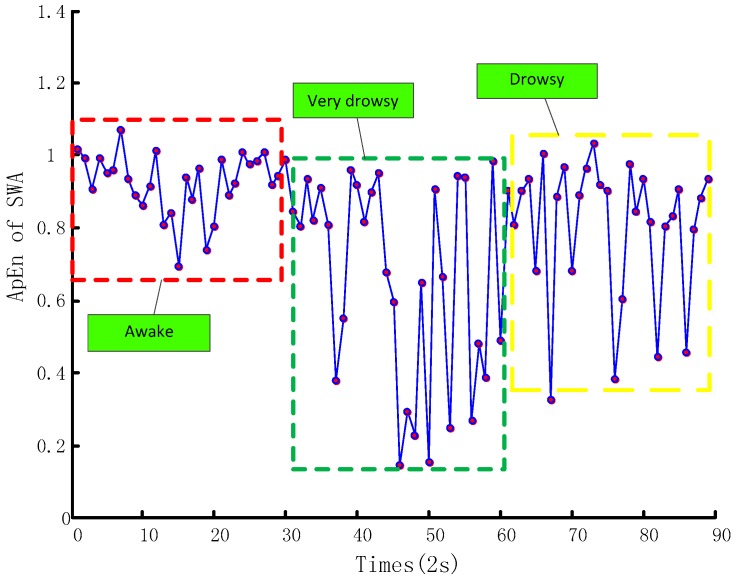
SWA ApEn distribution.

**Figure 7:**
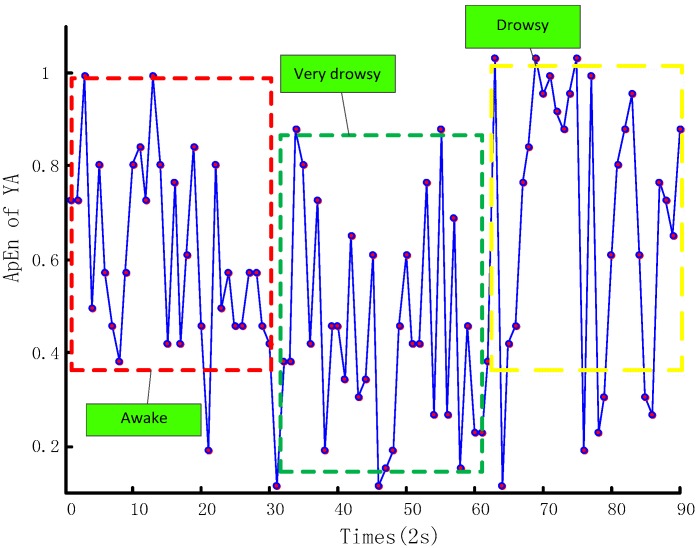
YA ApEn distribution.

Figure 6 demonstrates the ApEn distribution of the SWA samples’ time series calculated according to Equation (1). The vertical coordinate represents the ApEn of SWA within a short period of time (two seconds) and the horizontal coordinate is the time of three samples, each lasting one minute. From this chart, we can see that when the driver is at different fatigue levels, the ApEn of SWA time series have different distribution features, explaining the meaning of exploring SWA fatigue features. Similarly, Figure 7 shows similar YA information. These two figures demonstrate that ApEns of SWA and YA time series can fully represent drivers’ operation behavior features, and thus identify drivers’ fatigue levels.

We conducted a three-level fatigue identification test on 212 samples from six subjects, using SWA and YA ApEn. The experiment, as demonstrated in Table 3, obtained an accuracy of 88.02%. Compared with previous research [15], the accuracy only rises by 0.32%, but our data are retrieved from a real driving environment, so the engineering applicability of this system is actually enhanced.

### 4.4. Comparison between Testing Results by Relevant Methods

To prove the superiority of the proposed method in real road driving conditions, we compared the identification accuracy of driver operation parameters by different testing methods (results are given in Table 4). Literature [14] makes statistical feature analysis of SWA time series under lab conditions, and fatigue level identification with the fisher classifier was accurate to 82%. Previous research [15], with the same statistical features, uses the SVM classifier, improving its accuracy to 87.7%. Other studies [16,17] based on SWA information obtained in real road driving conditions, and using ApEn features and a simple classifier, achieved an averaged accuracy of 82.07% and 78.01%, respectively. Based on these results, this paper introduces in a new type of data, the yaw angle of the vehicle, and achieves an accuracy of 88.02%.

## 5. Discussion

The method in this paper uses SWA and YA information to identify the fatigue state of a driver, on the grounds that drivers’ physical statuses have a direct reflection on their operation behaviors. The turning of the steering wheel and the yaw of the vehicle are the most sensitive and frequent. Fatigue identification occurs at three levels: “awake”, “drowsy” and “very drowsy”, boasting a performance of 88.02% accuracy during approximately 15 h of driving on real road. The fatigue identification system using SWA and YA time series enjoys higher robustness and reliability, which has been proven by the real road driving test, instead of a lab simulation test. Meanwhile, this system has a very low rate of false alarms of “drowsy” (7.50%) and “very drowsy” (7.91%), as shown in Table 3. It is robust because its evaluation criteria, shown in Table 1, have acquired consistent results in evaluation by experts and in others’ works, which are facial video based rather than SWA information based. This set of criteria is a combination of drivers’ facial expressions, head positions and physical statuses, whose universal applicability in driver fatigue evaluation ensures the robustness of this identification system. 

## 6. Conclusions and Future Works

This paper presents a fatigue detection system using SWA and YA information obtained by fixed sensors. The evaluation of fatigue state is made with the combined information of drivers’ facial expressions, head positions and physical states, as recorded in videos and with the experts’ agreed judgments taken as standards. During the detection, ApEn features are used to make the judgment with the BP neural network classifier. This method is suitable for detection of offline SWA and YA samples, and fatigue detection at three levels (“awake”, “drowsy” and “very drowsy”). It receives an overall accuracy of 87.21% in detection. Previous work [26] has proven that SWA, in combination with vehicles’ lateral positions and yaw angles, will improve accuracy in fatigue identification in a lab simulation environment. Inspired by this, to combination of more drivers’ operational features, and vehicle status parameters, in fatigue identification to improve the accuracy is the direction of our future work.

## Figures and Tables

**Table 1:** Driver fatigue level criteria after evaluation.

Driving Status	Fatigue Label	Features
Awake	1	The head stays upright, and facial expressions are rich. Attentive to the environment. Eyes open widely and blink quickly and eyeballs move actively.
Drowsy	2	Attention to the outside world decreases. Drivers make gestures like scratching faces, shaking head, winking, swallowing, sighing, deep breathing, and yawning. Eyes tend to close, blink slowly with less eyeball activity.
Very drowsy	3	Eyes close further with eyelids becoming heavier. Eyes are closing for a longer time. Drivers may nap, nod, slant their heads, and then lose the ability to drive.

**Table 2:** Sample database.

Serial No. of Subjects	Number of Samples	Fatigue Level
910_002	34	(0,1)
910_004	48	(0,1,2)
911_003	29	（0,1）
912_007	24	(0,1)
913_002	23	(0,1)
913_004	54	(0,1,2)

**Table 3:** Detection results distributed on confusion matrix for three fatigue levels “0”, “l” and “2”.

		Detection Results		
		“Awake” (Level 0)	“Drowsy” (Level 1)	“Very drowsy” (Level 2)
Expert classification	“Awake” (Level 0)	92.50%	7.50%	7.00%
	“Drowsy”(Level 1)	7.50%	84.60%	14.11%
	“Very drowsy”(Level 2)	0.00%	7.90%	78.89%
Samples		112	72	28

**Table 4:** Results comparison between five methods.

Experiment Data	Method	Average Correct Rate (%)
SWA for laboratory driving conditions [14]	Statistical Feature + Fisher	82.00
SWA for laboratory driving conditions [15]	Statistical Feature + SVM	87.70
SWA for real driving conditions [16]	ApEn Feature + Designed model	82.07
SWA for real driving conditions [17]	ApEn Feature + Designed model	78.01
SWA and YA for real driving conditions (presented in this paper)	ApEn Feature + BP NN	88.02

## References

[1] Sahayadhas A., Sundaraj K., Murugappan M. (2012). Detecting driver drowsiness based on sensors: Review. Sensors.

[2] Williamson A., Lombardi D.A., Folkard S., Stutts J., Courtney T.K., Connor J.L. (2011). The link between fatigue and safety. Accid. Anal. Prev..

[3] Connor J.L. (2009). The role of driver sleepiness in car crashes: A review of the epidemiological evidence. Drugs, Driving and Traffic Safety.

[4] Khushaba R.N., Kodagoda S., Lal S., Dissanayake G. (2011). Driver drowsiness classification using fuzzy wavelet-packet-based feature-extraction algorithm. IEEE Trans. Biomed. Eng..

[5] Chai R., Naik G., Nguyen T.N., Ling S., Tran Y., Craig A. (2016). Driver fatigue classification with independent component by entropy rate bound minimization analysis in an eeg-based system. IEEE J. Biomed. Health Inform..

[6] Chai R., Ling S.H., San P.P., Naik G.R., Nguyen T.N., Tran Y. (2017). Improving eeg-based driver fatigue classification using sparse-deep belief networks. Front. Neurosci..

[7] Huang K.C., Huang T.Y., Chuang C.H., King J.T., Wang Y.K., Lin C.T. (2016). An eeg-based fatigue detection and mitigation system. Int. J. Neural Syst..

[8] Craig A., Tran Y., Wijesuriya N., Nguyen H. (2012). Regional brain wave activity changes associated with fatigue. Psychophysiology.

[9] Picot A., Charbonnier S., Caplier A. (2012). On-line detection of drowsiness using brain and visual information. IEEE Trans. Syst. Man Cybern. Part A.

[10] Zhang C., Wang H., Fu R. (2014). Automated detection of driver fatigue based on entropy and complexity measures. IEEE Trans. Intell. Transp. Syst..

[11] Correa A.G., Orosco L., Laciar E. (2014). Automatic detection of drowsiness in EEG records based on multimodal analysis. Med. Eng. Phys..

[12] Jung S.J., Shin H.S., Chung W.Y. (2014). Driver fatigue and drowsiness monitoring system with embedded electrocardiogram sensor on steering wheel. IET Intell. Transp. Syst..

[13] Mandal B., Li L., Wang G.S., Lin J. (2016). Towards detection of bus driver fatigue based on robust visual analysis of eye state. IEEE Trans. Intell. Transp. Syst..

[14] Zhang X., Cheng B., Feng R. (2010). Real-time detection of driver drowsiness based on steering performance. J. Tsinghua Univ..

[15] Qu X., Cheng B., Lin Q., Li S. (2013). Drowsy driving detection based on driver’s steering operation characteristics. Autom. Eng..

[16] Li Z.J., Li S.E., Li R.J., Cheng B., Shi J.L. (2017). Driver fatigue detection using approximate entropic features of steering wheel angle from real driving data. Int. J. Robot. Autom..

[17] Li Z.J., Li S.E., Li R.J., Cheng B., Shi J.L. (2017). Online Detection of Driver Fatigue Using Steering Wheel Angles for Real Driving Conditions. Sensors.

[18] Wang J.Q., Lu M., Li K.Q. (2010). Characterization of longitudinal driving behavior by measurable parameters. Transp. Res. Rec..

[19] Wang J.Q., Zhang L., Zhang D.Z. (2013). An adaptive longitudinal driving assistance system based on driver characteristics. IEEE Trans. Intell. Transp. Syst..

[20] Wang J.Q., Li S.E., Zhang Y. (2015). Longitudinal collision mitigation via coordinated braking of multiple vehicles using model predictive control. Integr. Comput. Aided Eng..

[21] Fukuda J., Akutsu E., Aoki K. (1995). Estimation of driver’sdrowsiness level using interval of steering adjustment for lane keeping. JSAE Rev..

[22] Eskandarian A., Mortazavi A. Evaluati on of a smart algorithm for commercial vehicle driver drowsiness detection. Proceedings of the 2007 IEEE Intelligent Vehicles Symposium.

[23] Bittner R., Hana K., Pousek L. Detecting of fatiguestates of a car driver. Proceedings of the International Symposium on Biological and Medical Data Analysis.

[24] Krajewski J., Golz M., Sommer D. Detecting sleepy drivers by pattern recognition based analysis of steering wheel behavior. Proceedings of the Fifth International Driving Symposium on Human Factors in Driver Assessment, Training and Vehicle Design.

[25] Wierwille W.W., Ellsworth L.A. (1994). Evaluation of driver drowsiness by trained raters. Accid. Anal. Prev..

[26] Qu X., Chen B. (2012). Detection of Driver Drowsiness Based on Steering Operation and Vehicle State. Master’s Thesis.

[27] Pincus S.M., Goldberger A.L. (1994). Physiological time-series analysis: What does regularity quantify?. Am. J. Physiol..

[28] Richman J.S., Moorman J.R. (2000). Physiological time-series analysis using approximate entropy and sample entropy. Am. J. Physiol. Heart Circ. Physiol..

[29] Zhang Z., Zhou Y., Chen Z.Y., Tian X.H., Du S.H., Huang R.M. (2013). Approximate entropy and support vector machines for electroencephalogram signal classification. Neural Regen. Res..

[30] Lin L., Chu F.L. (2011). Approximate entropy as acoustic emission feature parametric data for crack detection. Nondestruct. Test. Eval..

[31] He Y., Zhang X. (2012). Approximate entropy analysis of the acoustic emission from defects in rolling element bearings. J. Vib. Acoust..

[32] Kumar Y., Dewal M.L., Anand R.S. (2014). Epileptic seizure detection using DWT based fuzzy approximate entropy and support vector machine. Neurocomputing.

[33] Yentes J.M., Hunt N., Schmid K.K., Kaipust J.P., McGrath D., Stergiou N. (2013). The appropriate use of approximate entropy and sample entropy with short data sets. Ann. Biomed. Eng..

[34] Pincus S.M. (1991). Approximate entropy as a measure of system complexity. Proc. Natl. Acad. Sci. USA.

[35] Cao X., Zhu D., Yang S.X. (2016). Multi-auv target search based on bio inspired aerodynamics model in 3-d underwater environments. IEEE Trans. Neural Netw. Learn. Syst..

[36] Chu Z., Zhu D., Yang S.X. (2016). Observer-based adaptive neural network trajectory tracking control for remotely operated vehicle. IEEE Trans. Neural Netw. Learn. Syst..

[37] Ni J., Li X., Hua M., Shen J. (2016). Bio inspired neural network based q-learning approach for robot path planning in unknown environments. Int. J. Robot. Autom..

